# Wider window, easier access: optimizing Leksell Vantage positioning for posterior fossa stereotactic biopsy

**DOI:** 10.1007/s00701-026-06774-x

**Published:** 2026-01-27

**Authors:** Chiara Barbesino, Andrea Bianconi, Pietro Fiaschi, Concetta Viola, Gianluigi Zona, Paolo Merciadri

**Affiliations:** 1 Department of Neuroscience, Rehabilitation, Ophthalmology, Genetics, Maternal and Child Health (DINOGMI), University of Genova, Genoa, Italy; 2 Department of Neurosurgery, IRCCS Azienda Ospedaliera Metropolitana di Genova, Genoa, Italy

**Keywords:** Stereotactic biopsy, Posterior fossa, Leksell® Vantage™

## Abstract

**Purpose:**

In Neurosurgery, stereotactic biopsy represents the main minimally invasive surgical technique to target deep and hard-to-reach brain sites that are not accessible by traditional surgical methods. The best trajectory for biopsy needle insertion is planned on the basis of CT or MRI studies. According to the relevant literature, although biopsy of the posterior cranial fossa with Leksell® Vantage™ Head Frame is possible, it involves several technical and clinical challenges. We describe an alternative configuration of the Leksell® Vantage™ system that addresses these limitations in the management of posterior cranial fossa lesions.

**Methods:**

At our Department (IRCCS Ospedale Policlinico San Martino in Genoa) we have used Leksell® Vantage™ stereotactic system together with Medtronic StealthStation™ S8 planning software to perform a series of seven stereotactic biopsies in the occipital or cerebellar region by rotating the stereotactic helmet 180° on the axial plane, resulting in an inversion of both the anteroposterior and lateral–lateral axes.

**Results:**

We demonstrate that stereotactic posterior fossa biopsies can be performed with helmet rotation, without additional procedural complications. No similar approach has previously been described in the literature.

**Conclusion:**

This approach significantly enhances access to the posterior cranial fossa and occipital lesions. This optimisation improves manoeuvrability, provides a more advantageous viewing angle for the needle-biopsy trajectory and allows for a less complex preoperative planning. The approach remains minimally invasive and is generally compatible with execution under conscious sedation.

## Introduction

In the domain of neurosurgery, stereotactic biopsy represents the prevailing minimally invasive surgical technique employed to access deep cerebral regions that are otherwise challenging to reach by conventional methods. In order to maximise the accuracy of the technique, preoperative planning integrates advanced neuroimaging methods, such as computed tomography (CT), magnetic resonance imaging (MRI) and positron emission tomography (PET), with a stereotactic system that allows for precise navigation to the target. The principal indications for stereotactic biopsy are as follows:

- The presence of suspected primary or metastatic neoplastic lesions.
- The presence of suspected inflammatory or infectious lesions.
- Neurodegenerative diseases which necessitate histological analysis of brain tissue.

Biopsies of the posterior cranial fossa remain technically complex due to the narrow surgical corridor and vascularity of the area.

The Leksell® Vantage™ stereotactic system (*Elekta AB, Stockholm, Sweden*) [2] in combination with modern planning platforms such as the Medtronic StealthStation™ S8 (*Medtronic Navigation, Inc., Louisville, Colorado*) [5] is routinely used in clinical practice at our centre.

Despite the system's ergonomic benefits, its current configuration imposes limitations on the width of the operational window available for posterior trajectories. Recent publications have demonstrated the feasibility of such approaches to the posterior cranial fossa with this system, however they generally require general anaesthesia and a more demanding preoperative planning and helmet positioning to avoid mechanical conflicts between the arch and the frame.

This article delineates an alternative configuration of the Leksell® Vantage™ system that partially mitigates these limitations and facilitates the management of lesions located in the posterior cranial fossa and occipital lobes.

## Methods

The Department of Neurosurgery at the IRCCS Policlinico San Martino Hospital in Genoa, Italy, has recently undertaken a series of seven stereotactic biopsies in the posterior cranial fossa. These procedures have incorporated modifications to the standardised use of the Leksell® Vantage™ system.

**Fig. 1:**
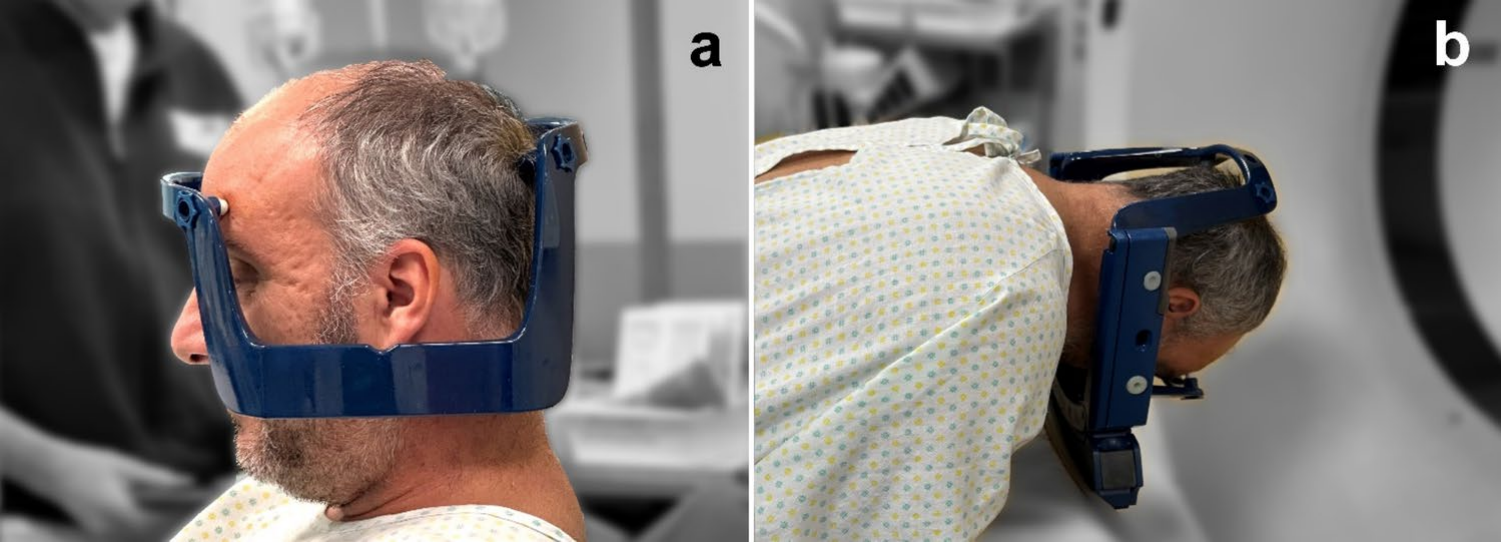
**a** Helmet positioning with inversion of the major axis in the anteroposterior direction. **b** Patient in prone position during CT scan

The procedure begins with the positioning of the stereotactic head frame following the administration of local anaesthesia. The helmet is rotated 180° on the axial plane, resulting in an inversion of both the anteroposterior and lateral–lateral axes (see Fig. 1a).

Subsequently, a contrast-enhanced cranial CT scan is performed to acquire the targeting. The patient is placed in the prone position (see Fig. 1b).

Medtronic StealthStation™ S8 software necessitates the CT scanner to be configured as if the examination is being conducted with the patient in the supine position. Otherwise, the software automatically rotates the images with the nose pointing upwards, making it impossible to register the stereotactic head frame in both automatic and manual mode.

Subsequently, CT images are processed (and possibly merged with MRI or PET images) to define the target coordinates and the desired trajectory.

**Fig. 2:**
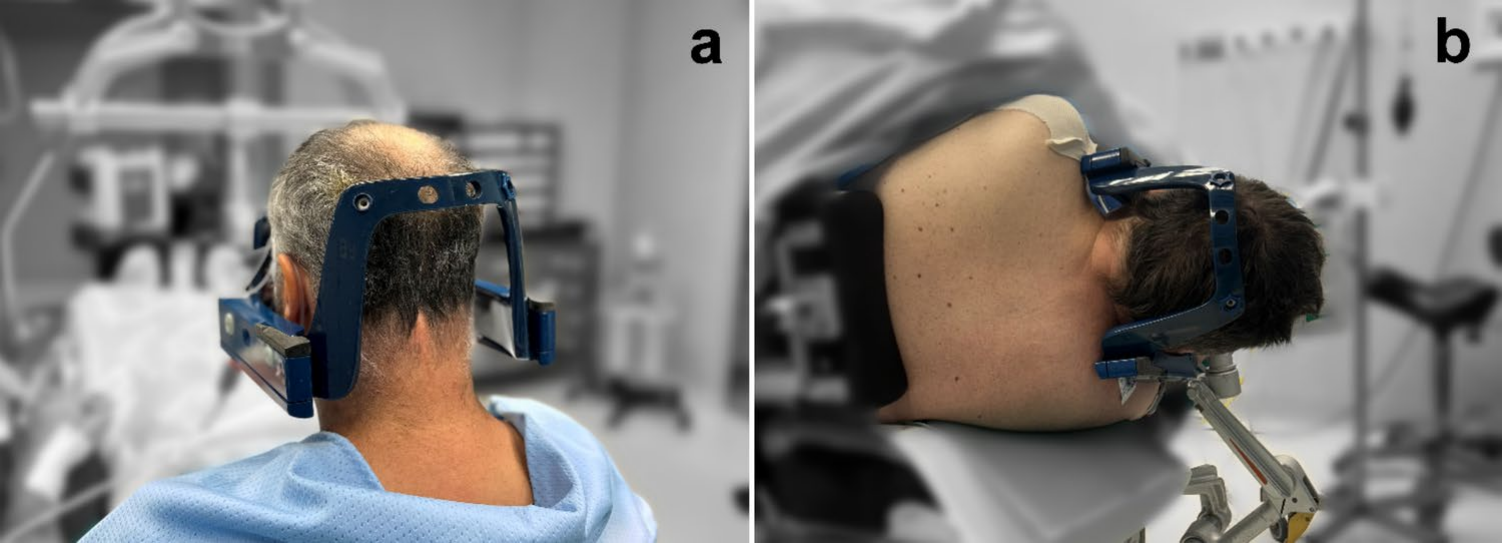
Patient in the operating theatre in (**a**) the semi-sitting position and (**b**) the right lateral decubitus position

With the exception of one case in which the patient was placed in a prone position under general anaesthesia (mainly due to poor cooperation), the procedure was performed in a semi-sitting or lateral decubitus position, allowing it to be conducted under local anaesthesia with conscious sedation (see Fig. 2a, b).

When setting and mounting the stereotactic arc system, it is imperative to acknowledge that the ‘lateral left’ and ‘lateral right’ configurations are contingent on the helmet's orientation rather than the patient's anatomical structure.

Finally, following the administration of local anaesthesia, Salcman twist drill (*Elekta AB, Stockholm, Sweden*) is used and the biopsy needle is inserted percutaneously and advanced along the planned trajectory. We routinely use the Sedan side-cutting biopsy needle kit (*Elekta Instrument AB, Stockholm, Sweden*), but other needles for core or aspiration biopsy are available depending on the lesion characteristics and the surgeon’s preference. As a standard practice, postoperative CT is used to verify correct targeting and the absence of minor intracranial complications.

A systematic search of PubMed using the term '(Leksell) AND (Vantage)' produced six articles. Three of these were excluded as they dealt with irrelevant topics, such as Gamma Knife radiotherapy or DBS.

The Leksell® Vantage™ stereotactic system can be used in performing posterior trajectories: the article “*Posterior fossa approaches using the Leksell Vantage frame with a virtual planning approach in a series of 10 patients: feasibility, accuracy, and pitfalls*”, published by Krüger et al. in 2022 [1] describes how the frame has to be positioned considering the entry point, lateral projection of the target and trajectory line. The frame is tilted anteriorly at a greater angle than the planned trajectory to allow for greater manoeuvrability. Patients were positioned in a sitting or semi-sitting position with the helmet attached to the Mayfield support.

Prilop et al. [3] published the paper “*Technical challenges and outcomes of stereotactic biopsies in the posterior fossa: experience with ZD-Inomed and Leksell Vantage frames*” in 2024. This paper describes stereotactic biopsies performed exclusively under general anaesthesia with the patient in the prone position.

In the paper “*Posterior Fossa Stereotactic Biopsy with Leksell Vantage Frame: Case Series and Review of Literature*” by Rowbottom et al. [4], an adjustment to frame positioning is described, involving a “lateral left” approach with slight rotation of the helmet, depending on the patient's characteristics and on whether there is any interference between the rear bar of the frame and the trajectory.

## Results

The literature review conducted suggests that posterior trajectories using the Leksell® Vantage™ system are feasible; however, published studies primarily emphasise optimisation of frame positioning according to target location to avoid mechanical collisions. Consequently, planning is more complex than for anterior approaches, and these procedures are usually performed under general anaesthesia.

**Fig. 3:**
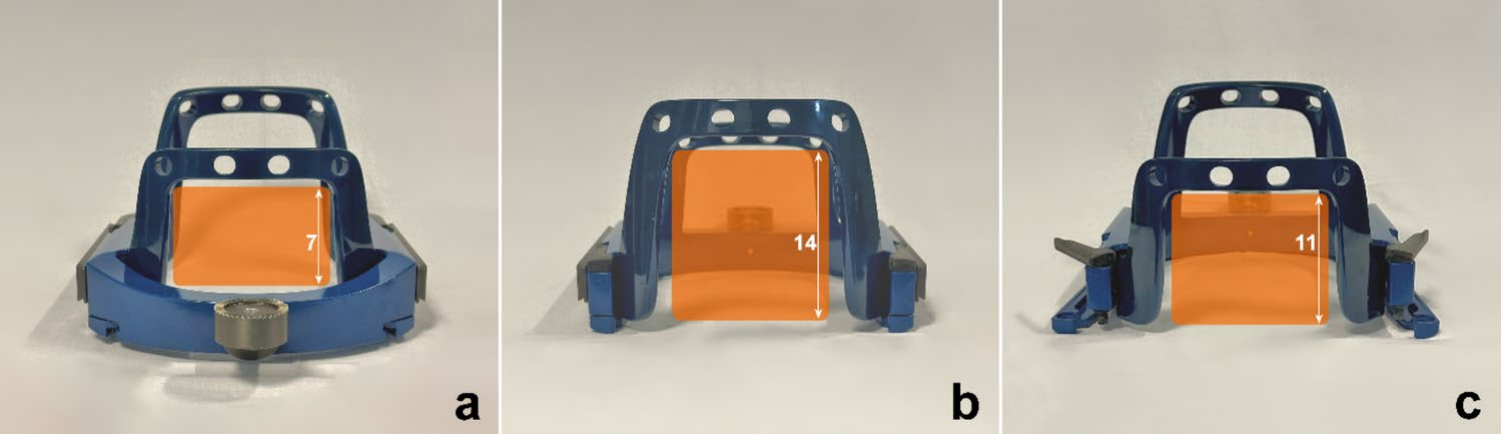
Leksell® Vantage head frame holder window: **a** standard positioning for posterior fossa procedures (11 × 7 cm, lateral × craniocaudal); **b** inverted helmet mounting (11 × 14 cm, lateral × craniocaudal); **c** inverted helmet holder (11 × 11 cm, lateral × craniocaudal)

**Fig. 4:**
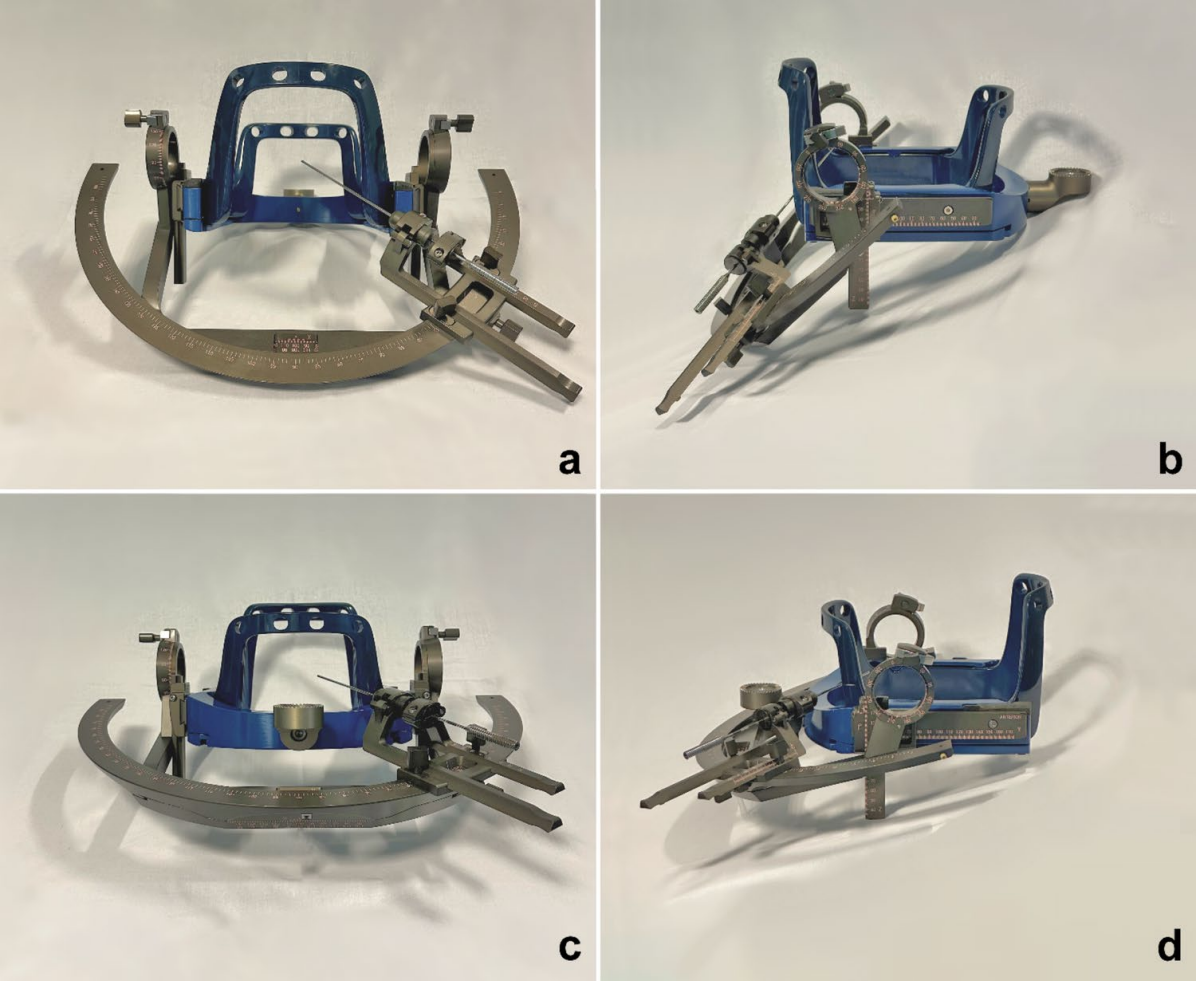
Inversion of the anteroposterior axis of the head frame permits a larger window, enabling a broader range of trajectories (**a**, **b**) compared with conventional positioning (**c**, **d**)

In our study, we demonstrate that such biopsies can be easily performed by rotating the stereotactic helmet 180° on the axial plane, resulting in an inversion of both the anteroposterior and lateral–lateral axes. This creates a larger operating space by moving posteriorly the anterior window, without increasing procedural complications (see Fig. 4). This approach significantly improves access to the posterior fossa. The size of the biopsy window is doubled, increasing from 11 × 7 cm to 11 × 14 cm, without interfering with the Mayfield support (see Fig. 3).

Careful planning based on CT was essential to achieve satisfactory and diagnostic results in the first seven biopsies performed (whose characteristics are shown in Table 1) (Fig. 4).

**Table 1:** Characteristics of patients who underwent stereotactic biopsy for posterior lesions at our institution. The following data are reported: age at the time of surgery, gender, location of the lesion, position in the operating theatre, type of anaesthesia, diagnostic histological sample and any complications

	Age (y)	Sex	Site	Position	Anaesthesia	Diagnostic	Complications
Patient 1	45	M	Right Occipital lobe	Semisitting	Local	Yes	None
Patient 2	57	M	Left Cerebellum	Right lateral decubitus	Local	Yes	None
Patient 3	60	M	Left Cerebellum	Right lateral decubitus	Local	Yes	None
Patient 4	33	F	Left Occipital lobe	Semisitting	Local	Yes	None
Patient 5	47	M	Right Occipital lobe	Semisitting	Local	No	None
Patient 5 #2	47	M	Left Cerebellum	Prone	General	Yes	None
Patient 6	63	M	Left Cerebellum	Semisitting	Local	Yes	Major haemorrhage

In the described sample (six patients, one of whom underwent the procedure twice due to inconclusive histological examination), the mean patient age was 50.83 years (range, 33–63 years), and there was a male prevalence. Surgical sites were the cerebellum and the occipital lobe, which were accessed primarily via the semi-sitting or lateral decubitus positions.

Most procedures were performed under local anaesthesia; only one required the prone position under general anaesthesia, mainly due to poor cooperation. Histological diagnosis was successfully achieved in all cases except one. We observed only a single complication, unfortunately a severe postoperative haemorrhage. The patient underwent an urgent surgical evacuation of the haemorrhagic lesion but died three days later due to a new massive haemorrhage involving a second supratentorial lesion (which had not been selected as the biopsy target because it was predominantly necrotic-cystic, with only a very thin peripheral ring of enhancement). The histological examination revealed they were rare urothelial carcinoma metastases.

## Discussion

Our study demonstrates that posterior fossa stereotactic biopsies can be performed using an alternative positioning of the Leksell® Vantage™ frame. This accelerates preoperative workflow (since no fine adjustments of the stereotactic frame positioning are required) and increases the working window and improves manoeuvrability during trajectory planning. This configuration enables safe access to lesions located in occipital lobe and posterior fossa, facilitating needle insertion while maintaining a minimally invasive approach.

This alternative frame positioning offers a feasible modification for posterior cranial fossa biopsies, enhancing surgical ergonomics and potentially reducing operative time while maintaining safety. The minimally invasive nature of stereotactic biopsy is preserved and the approach remains compatible with conscious sedation in most cases.

The main limitation of our study is the small number of patients, which restricts the generalisability of our findings. As with any stereotactic procedure, meticulous preoperative planning and adequate familiarity with the stereotactic hardware and software are essential for its successful execution.

Further studies with larger patient cohorts are required to confirm the reproducibility, feasibility and potential advantages of this frame positioning technique. A possible future technical development could involve the design of a dedicated support by Elekta, allowing the Mayfield adapter to be positioned in front of the patient’s face and providing a biopsy window measuring 11 × 11 cm. However, this configuration remains hypothetical and was not evaluated in the present study.

## Data Availability

Not applicable for this study.

## Declarations

### Declaration of Helsinki

The study was conducted in accordance with the declaration of Helsinki.
